# Cardiovascular effects and clinical outcomes in acute opioid toxicity: A case-control study from Port Said and Damietta Governorates Egypt

**DOI:** 10.1016/j.toxrep.2024.101756

**Published:** 2024-10-03

**Authors:** Heba Youssef Sayed, Rawan M. Ghaly, Amany A. Mostafa, Mohamed S. Hemeda

**Affiliations:** Department of Forensic Medicine and Clinical Toxicology, Faculty of Medicine – Port Said University, Egypt

**Keywords:** Opioid, Cardiac affection, ECG abnormalities, Cardiac enzymes, Echocardiography, Poison severity score

## Abstract

Substance abuse, particularly opioid intoxication, presents a significant public health challenge, leading to severe cardiovascular complications. This case-control study assessed the cardiac profile and clinical outcomes of 51 patients with confirmed acute opioid toxicity, compared to 51 control participants, in general hospitals across Port Said and Damietta governorates, Egypt. The study revealed that opioid-intoxicated patients exhibited significant cardiovascular abnormalities, including hypotension (39.2 %) and electrocardiogram (ECG) changes (72.5 %), with sinus bradycardia (51 %) being the most common. Additionally, echocardiographic abnormalities were found in 40 % of cases, with abnormal regional wall motion and valvular defects observed in several patients. Elevated levels of cardiac enzymes, such as Troponin-I and CK-MB, were significantly correlated with increased ICU stay length and higher mortality rates. The most common morbidities included coma (64.7 %) and shock (39.2 %). The study underscores the critical need for early cardiac assessment in opioid-intoxicated patients to predict clinical outcomes and guide therapeutic interventions.

## Highlights

• Opioid toxicity leads to significant cardiovascular abnormalities, especially sinus bradycardia.
• Elevated Troponin-I and CK-MB levels correlate with ICU stay length and mortality.
• Echocardiographic abnormalities are prevalent in opioid-intoxicated patients.
• Coma and shock are common morbidities in acute opioid intoxication cases.
• Early cardiac assessment is crucial for predicting outcomes in opioid toxicity.

## Graphical Abstract

**Figure:**
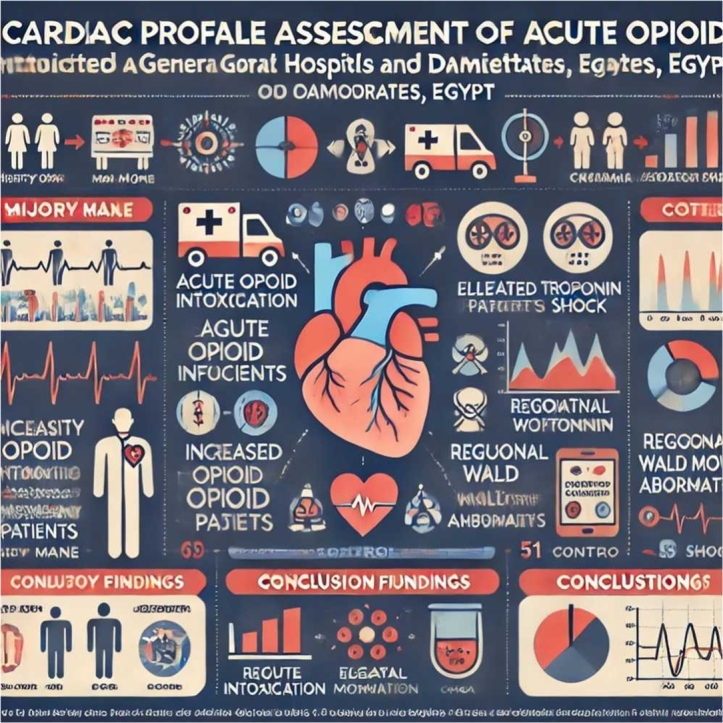

## 1 Introduction

Substance abuse remains a major public health problem around the world. With the existence of more effective medicines and higher numbers of drugs and their different combinations, there is a great challenge to prevent and treat drug abuse disorders. It requires a multi-faceted approach that includes education, prevention, treatment, and recovery support [1].

Although the mortality rate resulting from drug overdose is highly increasing, non-fatal overdoses have also been associated with renal dysfunction, aspiration pneumonia, heart and musculoskeletal problems, and cognitive impairment. Poisoning has serious consequences, including impairment and physical injury. Expenditure on treating substance abuse incidents has increased dramatically due to increases in overall emergency department visits, hospital admissions, and ICU admissions [1].

Drug overdose remains one of the leading causes of death, especially among young people in Europe. According to recent data, they are responsible for more than 3.4 % of all deaths in Europeans aged 15–39. The number of drug overdose deaths in the United States exceeds all expectations. According to the Centers for Disease Control and Prevention (CDC), drug overdoses are the leading cause of injury-related deaths in the United States and have more than tripled over the past 20 years. According to the National Center for Health Statistics (NCHS), that's more deaths than car crashes and homicides [2].

Toxicity in pediatrics is a significant public health issue in many countries, representing a major emergency in children. Toxicity with opiates or opioids is one of the most common toxicities among children in some regions of Iran. Opiate poisoning is reported to account for approximately 39 % of all childhood poisonings and 75 % of all childhood poisoning-related deaths in this region [3].

Between 2007 and 2014, a nationwide addiction survey found that Egypt, particularly Greater Cairo, has a greater rate of drug abuse than the rest of the world [4]. Overdoses caused by opioids that are deadly or non-fatal are extremely important public health issues. The likelihood of an overdose is influenced by the chemical used, the route of administration, and the user's health. The majority of overdose deaths are linked to the usage of opioids, especially heroin injections [2].

Synthetic analgesic tramadol has both opioid and non-opioid characteristics. Pharmacological dose forms that are both parenteral and oral. Overdoses and abuse of tramadol have increased recently in Iran. Tramadol's most frequent adverse effects are vomiting, dry mouth, nausea, dizziness, and sleepiness. The two most serious clinical side effects of therapeutic and toxic tramadol dosages are seizures and apnea [4].

As overdoses of opioids are on the rise, opioids will soon overtake all other drugs as the leading cause of overdose deaths. The number of heroin overdose deaths on a national level has increased by about 15 times since 1999. This is most likely because abuse of heroin is frequently mixed with fentanyl, an opioid that is 50–100 times more lethal than morphine. This has been theorized to be the cause of the ongoing increase in opioid-related fatalities despite the CDC's increasing awareness campaigns [5].

An increasing number of patients are being admitted to healthcare institutions with instances of overdose associated with opioids, which encompass both prescribed opioids, synthetically produced opioids and heroin; consequently, the critical care sector will be compelled to manage the most complex cases. Moreover, these hospital admissions may incur supplementary expenditures for both healthcare facilities and society at large, as this demographic may require escalating levels of sophisticated and costly multiorgan support [6].

The principal indicators of opioid toxicity encompass miosis, hallucinations, somnolence, emesis, respiratory depression, disorientation, myoclonic jerks, and varying degrees of diminished consciousness, which may manifest as drowsiness, stupor, or coma. The predominant cause of mortality is generally attributed to respiratory depression, although, in certain instances, it may stem from hypothermia. The most frequently documented signs and symptoms indicative of overdose morbidity include pulmonary complications such as edema and pneumonia, musculoskeletal problems such as rhabdomyolysis resulting from sustained pressure on musculature during comatose states, and renal impairment due to the breakdown of muscle tissue [7].

Opioid affects the cardiovascular system, with three major adverse cardiovascular complications, including hypotension, bradycardia, and QT prolongation, having been reported. The latter is believed to be one of the leading causes of cardiac arrest and life-threatening arrhythmias induced by opioid toxicity. However, the complex mechanism of opioid-induced QT interval prolongation suggests that, under particular and unpredictable circumstances or in conjunction with other drugs or diseases, opioids have an increased likelihood of causing ventricular tachycardia and sudden cardiac death [8].

The rise in opioid abuse has given rise to multiple unrecognized toxicity manifestations, particularly at young ages. Numerous clinical and experimental research that concerned about how opium affected the cardiovascular system found many negative effects. QT interval lengthening, bradycardia, torsade de pointes arrhythmia, and coronary artery disorders are the primary findings. The most frequent cardiac side effect of synthetic opioids, particularly methadone, appears to be QT prolongation. Heart complications related to opioids can increase the risk of death, so we study the cardiovascular profile of acute opioid-intoxicated patients [3].

## 2 Patients and methods

### 2.1 Ethical approval

The Ethical Research Committee of the Faculty of Medicine, Port Said University, accepted the entire research plan. (ERN number: (103) FCT 814a _001).

### 2.2 Study population

The study included 102 participants in the cases group who were previously healthy individuals with confirmed opioid toxicity (corresponding clinical symptoms and signs, and a positive urine toxicological screening) with any age of both genders. Patients are admitted to the emergency department (ER), inpatient department, intensive care unit (ICU), and toxicological laboratory at general hospitals in Port Said and Damietta governorate, Egypt. The control group included an equal number of the studied cases and matched in age and sex. They were not exposed to any poisonous substance, had no chronic illness or history of drug abuse, and obtained from relatives of cases who visited the hospital after giving written consent.

**Exclusion criteria**: Patients with any neurological disorders, patients with previous cardiac disease, diabetes, hypertension, alcohol abuse, and those who have a history of consuming many substances at the same time were excluded.

### 2.3 Patients consent

Before the participants were included in the study, written informed consent was obtained from all the participants or the legal guardian (in cases of children) before inclusion in the study, explaining the value of the research plus the procedures that were commenced.

### 2.4 Methods

All patients incorporated in this study were subjected to the following:

#### 2.4.1 Full history taking

• Demographic data: Age, sex, place of residence, job, degree of education, marital status, socioeconomic status, special habits of medical importance, such as smoking, and age of starting smoking. The delay time per hour (period between taking the substance and admission to the emergency department of a hospital). [5]
• Opioid type, estimated amount, form, method, and exposure mode.

#### 2.4.2 Examination based on grading of Poison Severity Score PSS

• Patients were classified according to the Poison Severity Score (PSS) into [6]:

➣ Grade 0 (None): asymptomatic with no symptoms or signs related to toxicity.
➣ Grade I (minor): mild symptoms and signs of toxicity
➣ Grade II (moderate): pronounced symptoms or signs of toxicity
➣ Grade III (severe): life-threatening symptoms or signs of toxicity
➣ Grade IV (Fatal): Fatal toxicity

#### 2.4.3 Cardiac assessment

• Local cardiac examination**:** Inspection, palpation, percussion, auscultation.
• Cardiac imaging techniques: ECG, which was done in all cases, and transthoracic echocardiography, which was done in some selected cases with severe symptoms or shocked patients.
• Cardiac enzyme measurement. Troponin I was done in selected cases (in severe cases or those with ECG ischemic changes), and Creatine kinase – myocardial band (CK-MB)

#### 2.4.4 Laboratory investigations

• Complete Blood Count,
• Arterial Blood Gases
• Serum Alt and AST
• Bun and Creatinine
• Serum Electrolytes (Na And K)
• Random Blood Glucose
• Urine Analysis (Rapid Drug Detection Kit In The Urine)

#### 2.4.5 Management parameters

##### 2.4.5.1 Therapeutic management data

This was done according to the American College of Medical Toxicology, emergency measures

• Management of airway
• Breathing,
• Circulation
• Decontamination
• Drug administration
• Finding an antidote,
• General supportive measures.

##### 2.4.5.2 Patients' outcome data

− -Morbidities (coma, seizures, sepsis).
− -ICU length of stay (days).
− -Duration of mechanical ventilation (days).
− -Recovery
− -Mortality.

##### 2.4.5.3 Admission

Patients are admitted to the toxicology department, where they get ongoing care and observation until they fully recover.

##### 2.4.5.4 - Referral

Cases **who were referred to other** poison control centers.

##### 2.4.5.5 - Lost follow-up

**Cases of people who** escaped from the hospital without a diagnosis or full course of treatment.

##### 2.4.5.6 Statistical analysis of data

Results were statistically analyzed by using a statistical package of social sciences (SPSS 26.0, IBM/SPSS Inc., Chicago, IL). Two types of statistical analysis were conducted using recent statistical methods, two types of statistical analysis were performed: Descriptive statistics: The descriptive statistics encompassed estimates for the summarization of continuous variables, represented by the mean (X), standard deviation (SD), median (Med), and range for data exhibiting skewness. The presentation of qualitative data was accomplished using frequency alongside percentage (%). Analytical or inferential statistics: This section comprised various statistical tests, including the Chi-Square test, which was utilized to compare two or more groups. The Fischer Exact test served as a correction for the Chi-Square test when over 25 % of the cells contained counts fewer than five in 2*2 contingency tables. The one-way analysis of variance (ANOVA) was employed to ascertain whether statistically significant differences exist among the means of three or more independent (unrelated) groups. The Kruskal-Wallis test functions as a statistical method for comparing two or more groups concerning a continuous or discrete variable. The Kruskal-Wallis test is occasionally referred to as the one-way ANOVA on ranks or the Kruskal-Wallis one-way ANOVA. The Binary Logistic regression model is classified within a broader category of statistical models known as generalized linear models. The distinguishing feature that sets binary logistic regression apart from alternative generalized linear models is the nature of the dependent (or outcome) variable, which in this context possesses two levels. The Mann-Whitney U test represents a nonparametric method that facilitates the comparison of two groups, conditions, or treatments without necessitating the assumption of normal distribution for the values. Level of significance (P-value): In all statistical analyses performed, the P-values associated with the test statistics denoted the significance threshold at which the null hypothesis (which posits no difference) was rejected, established at 0.05. Consequently, P-values ≥ 0.05 are deemed statistically non-significant, P-values < 0.05 are classified as significant, and P-values < 0.01 are regarded as highly important.

## 3 Results

Fifty-one of the acute opioids intoxicated patients were admitted to general hospitals of Port Said and Damietta governorates. The same number of a control group regarding sociodemographic data in this study (Table 1) revealed that most of the studied cases were males (94.1 %), their mean age was 32 (SD ±14), coming from urban areas (78.4 %), not working (58.8 %). As regards their education (39.2 %) have primary education, (23.5 %) have secondary education, (21.6 %) illiterate and (15.7 %) have university degree, more than half of them were single (64.7 %), more than half of the studied cases (68.6 %) have low socioeconomic status, most of them were smokers (84.3 %), their mean age when starting smoking was 19 (SD ± 3.8). Regarding the control group, they were matched in age and sex with study cases; most of them were working (70.6 %), (17.6 %) had university degrees, nearly half of them had average socioeconomic status (49 %), most of them were nonsmokers (86.3 %).

**Table (1):** Sociodemographic data among cases with confirmed opioid toxicity and control group.

		**Cases (n=51)**		**Control (n=51)**	
**Age Mean** ± **SD**		**32** ± **14**		**32** ± **14**	
		**N**	**%**	**N**	**%**
**Sex**	**Male**	**48**	**94.1**	**48**	**94.1**
	**Female**	**3**	**5.9**	**3**	**5.9**
**Residence**	**Rural**	**11**	**21.6**	**16**	**31.4**
	**Urban**	**40**	**78.4**	**35**	**68.6**
**Occupation**	**Working**	**21**	**41.2**	**36**	**70.6**
	**Not working**	**30**	**58.8**	**15**	**29.4**
**Education**	**None/illiterate**	**11**	**21.6**	**9**	**17.6**
	**Primary**	**20**	**39.2**	**12**	**23.5**
	**Secondary**	**12**	**23.5**	**9**	**17.6**
	**University**	**8**	**15.7**	**21**	**41.2**
**Marital Status**	**Single**	**33**	**64.7**	**21**	**41.2**
	**Married**	**15**	**29.4**	**28**	**54.9**
	**Divorced**	**2**	**3.9**	**2**	**3.9**
	**Widow**	**1**	**2**	**–**	**–**
**Socio-economic Status**	**Low**	**35**	**68.6**	**17**	**33.3**
	**Average**	**14**	**27.5**	**25**	**49**
	**High**	**2**	**3.9**	**9**	**17.6**
**Smoking Status**	**Smoker**	**43**	**84.3**	**7**	**13.7**
	**Non-smoker**	**8**	**15.7**	**44**	**86.3**
**Age of smoking initiation Mean**± **SD**		**19** ±**3.8**		**20** ±**3.9**	

Results showed significant associations with occupation (not working participants are more likely to be drug abusers), education (participants who have primary education are more likely to be drug abusers), marital status (not married participants are more likely to become drug abusers), socioeconomic status (participants with low socioeconomic status are more likely to be drug abusers), smoking status (smokers are more likely to be drug abusers). (Table 2)

**Table (2):** Independent t-test, fisher’s exact test, and Chi-square test for associations between sociodemographic characteristics among cases of confirmed opioid toxicity and control group.

		**Cases (n=51)**		**Control (n=51)**		**P-value**
**Age** Mean± SD		32 ± 14		32 ± 14		1.000^a^
		N	%	N	%	
**Sex**	Male	48	91.4	48	91.4	1.000^b^
	Female	3	5.9	3	5.9	
**Residence**	Rural	11	21.6	16	31.4	0.262^c^
	Urban	40	78.4	35	68.6	
**Occupation**	Working	21	41.2	36	70.6	**0.003**^c^
	Not working	30	58.8	15	29.4	
**Education**	None/illiterate	11	21.6	9	17.6	**0.037**^c^
	Primary	20	39.2	12	23.5	
	Secondary	12	23.5	9	17.6	
	Tertiary	8	15.7	21	41.2	
**Marital status**	Married	15	29.4	28	54.9	**0.009**^c^
	Unmarried	36	70.6	23	45.1	
**Socioeconomic status**	Low	35	68.6	17	33.3	**<0.001**^c^
	Average	14	27.5	25	49	
	High	2	3.9	9	17.6	
**Smoking status**	Smoker	43	84.3	7	13.7	**< 0.001**^c^
	Nonsmoker	8	15.7	44	86.3	
**Age of smoking initiation** Mean± SD		19± 3.8		20 ± 3.9		0.522^a^

P value > 0.05 is not significant, P value ≤ 0.05 is significant, P value ≤ 0.01 is very substantial, and P value ≤ 0.001 is highly important.

a Independent T-Test,

b Fisher’s Exact Test,

c Chi-Square Test

Regarding the type of opioid abused and route of intoxication, results revealed that more than half the cases abused opiates as a natural form of opioids (60.8 %). In comparison (37.3 %) were intoxicated with Tramadol (Table 3). The percentage of route of intoxication was equal among cases regarding ingestion and inhalation (37.3 %). In most cases, the mode of toxicity was addicting overdose (86.3 %).

**Table (3):** Frequency & percentage of type of opioid abuse among cases with confirmed opioid toxicity.

**Type of Opioid Abuse**		**Frequency (N=51)**	**Percentage**
**Substance abused**	**Tramadol**	19	37.3
	**Opiates**	31	60.8
	**Both**	1	2
**Route of intoxication**	**Ingestion**	19	37.3
	**Inhalation**	19	37.3
	**Injection**	12	23.5
	**Ingestion & inhalation**	1	2
**Mode of toxicity**	**Accidental**	6	11.8
	**Addict overdose**	44	86.3
	**Intentional**	1	2

Results revealed a significant association between opiates and male sex and low socioeconomic status, tramadol and unemployment, and being single. (Table 4)

**Table (4):** One-way ANOVA & Chi-square tests for associations between sociodemographic characteristics and type of opioid abuse among cases with confirmed opioid toxicity.

**Sociodemographic Characteristics**		**Substance Abused**								**P-value**
		**None**		**Tramadol**		**Opiates**		**Both**		
**Age**	Mean ± SD	32 ± 14								0.277^a^
		**N**	**%**	**N**	**%**	**N**	**%**	**N**	**%**	
**Sex**	Male	48	94.1	17	89.5	30	96.8	1	100	0.754^b^
	Female	3	5.9	2	10.5	1	3.2	–	–	
**Occupation**	Working	36	70.6	7	36.8	13	41.9	1	100	**0.015**^b^
	Not working	15	29.4	12	63.2	18	58.1	–	–	
**Education**	None/illiterate	9	17.6	5	26.3	6	19.4	–	–	0.222^b^
	Primary	12	23.5	7	36.8	12	38.7	1	100	
	Secondary	9	17.6	3	15.8	9	29	–	–	
	Tertiary	21	41.2	4	21.1	4	12.9	–	–	
**Marital status**	Married	28	54.9	4	21.1	11	35.5	–	–	**0.043**^b^
	Not married	23	54.1	15	78.9	20	64.5	1	100	
**Socioeconomic status**	Low	17	33.3	9	47.4	25	80.6	1	100	**0.003**^b^
	Average	25	49	8	42.1	6	19.4	–	–	
	High	9	17.6	2	10.5	–	–	–	–	

P value > 0.05 not significant, P value ≤ 0.05 significant, P value ≤ 0.01 very significant, P value ≤ 0.001 highly significant

a One Way ANOVA Test,

b Chi-Square Test

Results revealed an association between smoking status and both opioid and tramadol abuse; mostly, who was an opioid abuser was also a smoker. (Table 5)

**Table (5):** One-way ANOVA test for associations between smoking and type of opioid abused.

		**Substance Abused**										**P-value**
		**None**		**Tramadol**		**Opiates**			**Both**			
		**N**	**%**	**N**	**%**	**N**	**%**		**N**		**%**	
**Smoking Status**	Smoker	7	13.7	12	63.2	30	96.8		1		100	**<0.001**^b^
	Nonsmoker	44	86.3	7	36.8	1		3.2	–	–		
**Age of smoking initiation** Mean ±SD			19.3± 3.8									0.761^a^

P value ≤ 0.05 significant, P value ≤ 0.01 very significant, P value ≤ 0.001 highly significant

hi-Square Test

a One Way ANOVA Test

b C P value > 0.05 not significant,

Regarding the classification of study cases with confirmed opioid toxicity according to poison severity score, results revealed that the metabolic system, nervous system, cardiovascular system, and respiratory system are most commonly affected. Metabolic system changes were mostly moderate grade II with more pronounced acid-base disturbance. Regarding the nervous system, nearly half of the cases were moderate grade II (49 %) and presented with coma with an appropriate response to pain, agitation, seizures, and constricted nonreactive pupils. Respiratory system changes varied from minor grade I (mild dyspnea) (41.2 %), moderate grade II (hypoxia requiring extra oxygen) (31.4 %), and severe grade III (pulmonary edema, ARDS, pneumonia) (17.6 %). Regarding cardiovascular system severity of symptoms were (52.9 %) moderate symptoms, grade II (sinus tachycardia, sinus bradycardia, prolonged QRS, myocardial ischemia, more pronounced hypotension), only (7.8 %) presented with severe symptoms, grade III (myocardial infarction, shock, life-threatening ventricular arrhythmia) (Table 6).

**Table (6):** Clinical staging and severity of poisoning among cases with confirmed opioid toxicity.

**Clinical staging**	**Grade 0**			**Grade I**		**Grade II**		**Grade III**		**Grade IV**	
	**N**		**%**	**N**	**%**	**N**	**%**	**N**	**%**	**N**	**%**
**General and Metabolic Condition**	14	27.5		13	25.5	15	29.4	9	17.6	–	–
**Nervous System**	–	–		11	21.6	25	49	15	29.4	–	–
**Cardiovascular System**	9	17.6		11	21.6	27	52.9	4	7.8	–	–
**Respiratory System**	5	9.8		21	41.2	16	31.4	9	17.6	–	–
**Gastrointestinal System**	29	56.9		4	7.8	18	35.3	–	–	–	–
**Liver**	44	86.3		7	13.7	–	–	–	–	–	–
**Kidney**	45	88.2		–	–	6	11.8	–	–	–	–
**Skin**	39	76.5		5	9.8	5	9.8	2	13.9	–	–
**Muscular System**	42	82.4		3	5.9	6	11.8	–	–	–	–

Regarding cardiac profile assessment results, local examination showed that (39.2 %) of cases with confirmed opioid toxicity had hypotension n=20 (Tables 7), (13.7%) of cases had murmur n=7, (3.9 %) of cases had irregular pulse n=2, and (3.9 %) with absent peripheral pulsation n=2, compared with normal examination of the control group.

**Table (7):** Local cardiac examination results among cases with confirmed opioid toxicity and control groups.

**Cardiac Examination Signs**	**Cases (n=51)**		**Control (n=51)**	
	**N**	**%**	**N**	**%**
**Hypotension**	20	39,2	–	–
**Murmur**	7	13.7	–	–
**Irregular pulse**	2	3.9	–	–
**Absent peripheral pulsation**	2	3,9	–	–

Cardiac markers enzyme results revealed that cases with confirmed opioid toxicity had an increase in troponin levels with a mean (of 36.5) and CK-MB with a mean (of 48.9) (Table 8).

**Table (8):** Troponin (ng/ml) and CKMB (IU/L) measurements among cases with confirmed opioid toxicity and control group.

**Cardiac markers**		**Cases (n=51)** **Mean** ± **SD**		**Control (n=51)** **Mean** ± **SD**	
**Troponin (ng/ml)**		36.5 ±185.5		0.0078± 0.012	
**CK-MB (IU/L)**		48.9± 93.5		9.6± 3.07	
		**Cases (n=51)**		**Control (n=51)**	
		N	%	N	%
**Troponin-I (ng/ml)** **n=36**	Normal	8	22.2	36	70.6
	Abnormal	28	77.7	–	–
**CK-MB (IU/L)** **n=51**	Normal	25	49	51	100
	Abnormal	26	51	–	–

**Fig. (1):**
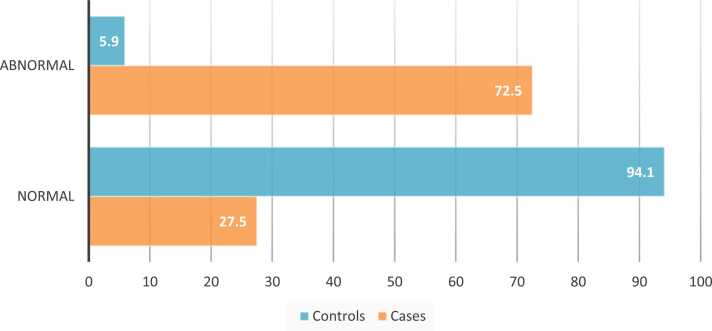
A bar chart showing ECG Findings among cases with confirmed opioid toxicity and control groups.

Out of 51 cases with confirmed opioid toxicity, 72.5 % had ECG changes compared to the control group. (Fig. 1).

**Fig. (2):**
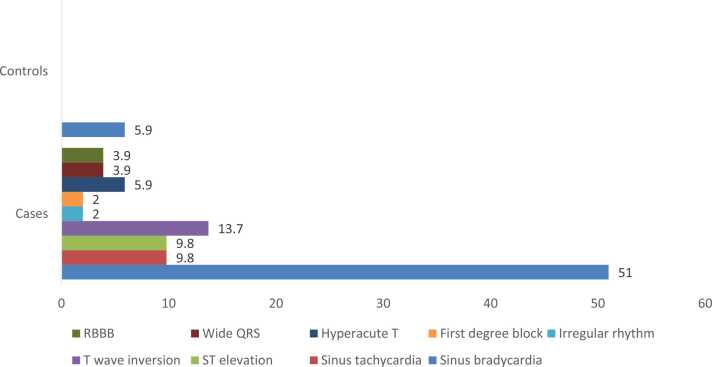
Column chart showing ECG abnormalities among cases with confirmed opioid toxicity and control groups.

ECG changes were more prominent among cases compared to the control group (Table 9); the most common ECG changes were sinus bradycardia (Fig. 2), more than half of the cases presented with sinus bradycardia 51 % n=26, 13.7 % presented with hyperacute T wave, 9.8 % with sinus tachycardia, 9.8 % with ST-segment elevation, 5.9 % presented with hyperacute T wave, 3.9 % with wide QRS complex, 3.9 % with RBBB right bundle branch block, 2 % presented with irregular rhythm, 2 % presented with first-degree heart block.

**Table (9):** Abnormalities of ECG among cases with confirmed opioid toxicity and control group.

**ECG Abnormalities**	**Cases (n=51)**			**Control (n=51)**		
	**N**		**%**	**N**	**%**	
1. **Sinus Bradycardia**	26		51	3	5.9	
2. **Sinus Tachycardia**	5		9.8	–	–	
3. **ST Elevation**	5	9.8		–		–
4. **T Wave Inversion**	7	13.7		–		–
5. **Irregular Rhythm**	1	2		–		–
6. **First Degree Heart Block**	1	2		–		–
7. **Hyperacute T-wave**	3	5.9		–		–
8. **Wide QRS complex**	2	3.9		–		–
9. **RBBB**	2	3.9		–		–
**Total**	51			51

**Fig. (3):**
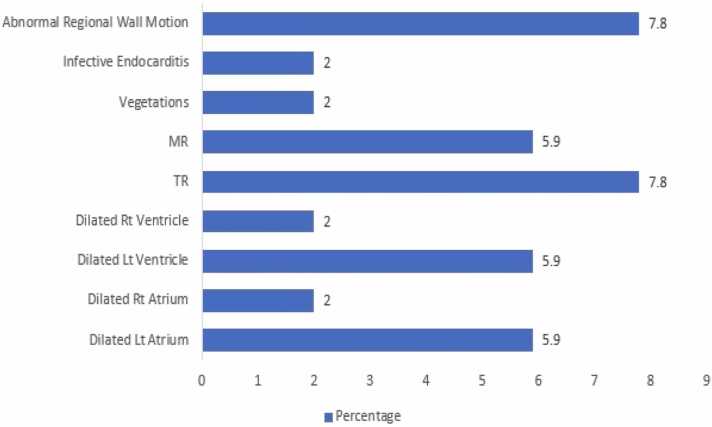
Bar chart of echocardiography abnormalities among cases with confirmed opioid toxicity and control group.

The most prominent echocardiography abnormalities were abnormal regional wall motion 7.8 % (Table 10), valvular abnormalities including tricuspid regurge TR 7.8 %, mitral regurge MR 5.9 %, dilated right ventricle and dilated right atrium 5.9 %, dilated left ventricle and dilated left atrium 2 %, infective endocarditis and vegetations 2 % (Fig. 3), (40 %) had abnormal ejection fraction while (60 %) had normal ejection fraction, and no abnormality was detected in the control group.

**Table (10):** Echocardiography abnormalities among cases with confirmed opioid toxicity and control group.

**Echocardiography Abnormalities**		**Cases (n=51)** **N %**		**Control (n=51)** **N %**	
**1-Abnormal regional wall motion**		4	7.8	–	–
**2-Infective endocarditis**		1	2	–	–
**3-Vegetations**		1	2	–	–
**4-MR**		3	5.9	–	–
**5-TR**		4	7.8	–	–
**6-Dilated Rt ventricle**		3	5.9	–	–
**7-Dilated Lt ventricle**		1	2	–	–
**8-Dilated Rt atrium**		3	5.9	–	–
**9-Dilated Lt atrium**		1	2	–	–
**Ejection Fraction n (%)** Cases (n= 20) Controls (n= 20)	Normal	13 (60)		20 (100)	
	Abnormal	7 (40)		–	
**Total**		20		20

Out of the sociodemographic characteristics, sex and smoking status (being male and a smoker) are more likely to be predictors of developing cardiac affection among cases with confirmed opioid toxicity (Table 11).

**Table (11):** Binary logistic regression test for predicting cardiac affection among study cases with confirmed opioid toxicity.

**Predictors**		**B**	**S. E**	**Odds Ratio**	**95 % CI**	**P-value**
**Age**		0.005	0.027	1.005	0.953, 1.060	0.856
**Sex**	Male			1		
	Female	−2.721	1.123	0.066	0.007, 0.595	**0.015**
**Residence**	Rural			1		
	Urban	−0.536	0.630	0.585	0.170, 2.011	0.395
**Occupation**	Not working			1		
	Working	0.370	0.604	1.447	0.443, 4.724	0.540
**Education**	None/illiterate			1		0.695
	Primary	0.258	0.755	1.294	0.295, 5.685	0.732
	Secondary	−0.072	0.800	0.930	0.194, 4.460	0.928
	Tertiary	0.944	0.895	2.569	0.444, 14.858	0.292
**Marital Status**	Unmarried			1		
	Married	1.130	0.647	3.095	0.872, 10.990	0.081
**Smoking Status**	Smoker			1		
	Nonsmoker	−3.064	0.770	0.047	0.010, 0.211	**<0.001**
**Socioeconomic Status**	Low			1		0.668
	Average	−0.462	0.650	0.630	0.176, 2.250	0.477
	High	−0.907	1.158	0.404	0.042, 3.906	0.434

P value > 0.05 not significant, P value ≤ 0.05 significant, P value ≤ 0.01 very significant

P value ≤ 0.001 is highly significant

The odds ratio (OR) is a quantifiable indicator of the strength of the association between an event and its corresponding exposure. B – This represents the unstandardized regression coefficient. It is evaluated as a multiple linear regression coefficient and can be elucidated in its interpretation. The Standard Error of the Regression quantifies the level of uncertainty regarding the precision of the predicted values of the dependent variable. A confidence interval, within the realm of statistics, denotes the likelihood that a population parameter will reside between a defined range of values for a specified proportion of instances.

Cardiac affection was highly associated with the type of substance abused, 60 % among opiate intoxicated patients and 30 % among tramadol-intoxicated patients. Other parameters are not significantly associated with developing cardiac affection (Table 12).

**Table (12):** Chi-square and mann-whitney u tests for associations between the type, route & mode of substance abuse and developing a cardiac affection.

		**Cardiac Affection**				**P-value**
		**Yes** **N %**		**No** **N %**		
**Type of substance abused (n=102)**	None	3	7.5	48	77.4	**<0.001**^a^
	Tramadol	12	30	7	11.3	
	Opiates	24	60	7	11.3	
	Tramadol and Opiates	1	2.5	–	–	
**Intoxication route (n=51)**	Ingestion	12	32.4	7	50	0.586^a^
	Inhalation	14	37.8	5	35.7	
	Injection	10	27	2	14.3	
	Ingestion and inhalation	1	2.7	–	–	
**Mode of toxicity (n=51)**	Accidental	3	8.1	3	21.4	0.360^a^
	Addict overdose	33	89.2	11	78.6	
	Intentional	1	2.7	–	–	
**Delay time (hours)**	Median (IQR)	5 (5)		4(4.5)		0.648^b^

P value > 0.05 not significant, P value ≤ 0.05 significant, P value ≤ 0.01 very significant, P value ≤ 0.001 highly significant

a Chi-Square Test,

b Mann-Whitney U Test

A significant association between CK-MB levels and the severity of CNS manifestations according to the poison severity score (Table 13).

**Table (13):** Kruskal Wallis and Chi-square tests for associations between cardiac profile and poisoning severity of central nervous system CNS among cases with confirmed opioid toxicity.

**Cardiac profile**		**Central Nervous System CNS Poisoning Severity**					**P-value**
		**Grade 0** **n (%)**	**Grade I** **n (%)**	**Grade II** **n (%)**	**Grade III** **n (%)**	**Grade IV** **n (%)**	
**Troponin (ng/ml)**	Mean (SD)	36.5 (185.5)					0.284^a^
	Median (IQR)	1 (2.26)					
**CK-MB (IU/L)**	Mean (SD)	48.9 (93.5)					**<0.001**^a^
	Median (IQR)	27 (28)					
**ECG Findings**	Normal	–	2 (16.7)	9 (29.1)	3 (13.1)	–	0.466^b^
	Sinus bradycardia	–	5 (41.8)	12 (38.7)	9 (39.2)	–	
	Sinus tachycardia	–	1 (8.3)	2 (6.5)	2 (8.7)	–	
	ST elevation	–	1 (8.3)	2 (6.5)	2 (8.7)	–	
	T wave inversion	–	–	2 (6.5)	5 (21.7)	–	
	Irregular rhythm	–	1 (8.3)	–	–	–	
	First-degree heart block	–	–	1 (3.1)	–	–	
	Hyperacute T	–	1 (8.3)	1 (3.1)	1 (4.3)	–	
	Wide QRS	–	–	2 (6.5)	–	–	
	RBBB	–	1 (8.3)	–	1 (4.3)	–	
**ECHO Findings**	Regional wall motion abnormalities	–	1 (25)	1 (25)	2 (15.4)	–	0.465^b^
	Dilated atrium	–	1 (25)	1 (25)	2 (15.4)	–	
	Dilated ventricle	–	1 (25)	–	3 (23.1)	–	
	TR	–	–	1 (25)	3 (23.1)	–	
	MR	–	1 (25)	–	2 (15.4)	–	
	Vegetations	–	–	1 (25)	–	–	
	Infective endocarditis	–	–	–	1 (7.6)	–	
**Ejection Fraction**	Mean (SD)	53.1 (13)					0.207^a^
	Median (IQR)	57.5 (22.7)					

a Kruskal Wallis Test,

b Chi-Square Test

**Fig. (4):**
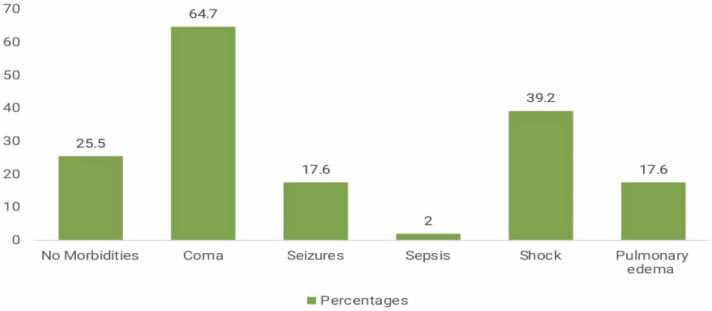
Bar chart showing morbidities among cases with confirmed opioid toxicity.

**Fig. (5):**
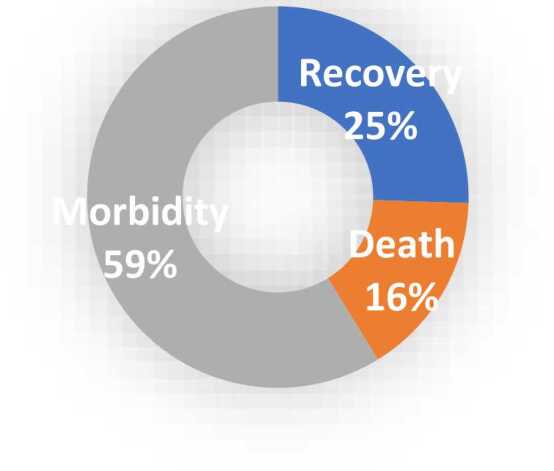
Pie chart showing clinical outcome among cases with confirmed opioid toxicity.

Regarding clinical outcome, results revealed that the most common morbidities among cases with confirmed opioid toxicity were CNS depression 64.7 % were in a coma, 17.6 % had seizures, only 2 % had sepsis,39.2 % had a shock, and 17.6 % developed non-cardiogenic pulmonary edema, (Table 14), (Fig. 4). The mean (SD) of ICU stay length per hour was 87.7 (49.8). In contrast, the duration of mechanical ventilation M.V. per hour among cases with confirmed opioid toxicity mean (SD) 72.1 (49.7) (Table 15), and more than half of intoxicated cases had morbidities at 59 %. In contrast, 25 % showed complete recovery, and 16 % died (Fig. 5).

**Table (14):** Morbidities among cases with confirmed opioid toxicity.

**Morbidities**	**Cases (n=51)** **N %**	
**Coma**	33	64.7
**Seizures**	9	17.6
**Sepsis**	1	2
**Shock**	20	39.2
**Non cardiogenic Pulmonary edema**	9	17.6
**Total**	38	74.5

**Table (15):** Mean and SD of ICU stay length per hour and duration of mechanical ventilation M.V. per hour among cases with confirmed opioid toxicity.

	**Cases (n=51)** **Mean (SD)**
**ICU Stay Length/hour**	87.7 (49.8)
**Duration of mechanical ventilation M.V. / hour**	72.1 (49.7)

There are significant correlations between Troponin-I, CK-MB, and ICU stay lengths (both correlations are weak and positive). An increase in the length of the ICU was associated with high levels of Troponin and CK-MB (Table 16).

**Table (16):** Correlations of cardiac markers with ICU stay length per hour among cases with confirmed opioid toxicity.

**Cardiac Markers**		**ICU Stay Length (hours)**
**Troponin (ng/ml)**	R	0.396
	p-value	**0.034**
**CK-MB (IU/L)**	R	0.386
	p-value	**0.006**

r: Spearman’s Correlation Coefficient

P value > 0.05 not significant, P value ≤ 0.05 significant, P value ≤ 0.01 very significant, P value ≤ 0.001 highly significant

Significant associations with Troponin-I (increased levels associated with mortality), CK-MB levels (increased levels associated with mortality), and ECG findings (sinus bradycardia and T wave inversion are associated with mortality) (Table 17).

**Table (17):** One-way ANOVA and Chi-square tests for associations between cardiac profile and clinical outcome.

**Cardiac profile**				**Clinical Outcome**		**P-value**
		**Death** **n (%)**	**Morbidity** **n (%)**		**Recovery** **n (%)**	
**Troponin (ng/ml)**	Mean (SD)	146.5 (376.8)	2.2 (2.8)		0.2 (0.6)	**0.036**^a^
**CK-MB** **(IU/L)**	Mean (SD)	59.1 (39.2)	126.4 (194.2)		13.2 (10.9)	**0.003**^a^
**ECG Findings**	Normal	1 (9.1)			61 (62.2)	**<0.001**^b^
	Sinus bradycardia	3 (27.3)			18 (18.4)	
	Sinus tachycardia	–			5 (5.1)	
	ST elevation	2 (18.2)			3 (3.1)	
	T wave inversion	3 (27.3)			4 (4.1)	
	Irregular rhythm	–			1 (1)	
	First-degree heart block	–			1 (1)	
	Hyperacute T	1 (9.1)			2 (2)	
	Wide QRS complex	–			2 (2)	
	RBBB	1 (9.1)			1 (1)	
**ECHO Findings**	Regional wall motion abnormalities	2 (28.6)			2 (10)	0.140^b^
	Dilated atrium	2 (28.6)			2 (10)	
	Dilated ventricle	1 (14.3)			3 (15)	
	Valvular abnormalities	1 (14.3)			5 (25)	
	TR	–			4 (20)	
	MR	1 (14.3)			2 (10)	
	Vegetations	–			1 (5)	
	Infective endocarditis	–			1 (5)	
**Ejection Fraction**	Mean (SD)	50.4 (14.6)	57.6 (11.9)		59.3 (8.9)	0.155^a^

P value > 0.05 not significant, P value ≤ 0.05 significant, P value ≤ 0.01 very significant, P value ≤ 0.001 highly significant

a One-way ANOVA test,

b Chi-Square Test

## 4 Discussion

Overdoses of opioids are on the rise; opioids will soon overtake all other drugs as the leading cause of overdose deaths. The number of heroin overdose deaths on a national level has increased by about 15 times since 1999 [5]. The rise in opioid abuse has given rise to multiple unrecognized toxicity manifestations, particularly at young ages. Numerous clinical and experimental research that concerned about how opium affected the cardiovascular system found many negative effects. QT interval lengthening, bradycardia, torsade de pointes arrhythmia, and coronary artery disorders are the primary findings. The most frequent cardiac side effect of synthetic opioids, particularly methadone, appears to be QT prolongation. Heart complications related to opioids can increase the risk of death, so we study the cardiovascular profile of acute opioid-intoxicated patients [3].

This study was carried out to assess the cardiac profile of acute opioids intoxicated patients admitted to general hospitals of Port Said and Damietta governorates. A case-control study design was selected. Study participants were classified into 2 groups: cases group with confirmed opioid toxicity and control group.

There were 102 study participants in this study, 51 cases with confirmed opioid toxicity and 51 controls matched with cases in age and sex; regarding history taking for sociodemographic data showed that most of the studied cases were males (94.1 %), their mean age was 32 (SD ±14), from urban areas (78.4 %), not working (58.8 %). As regards their education (39.2 %) have primary education, (23.5 %) have secondary education, (21.6 %) are illiterate and (15.7 %) have university degrees, more than half of them are single (64.7 %), (29.4 %) are married, (3.9 %) were divorced, and only (2 %) were widow, more than half of the studied cases (68.6 %) have low socioeconomic status. A significant association between opiate abuse and male sex and low socioeconomic status, tramadol and unemployment, and being single was recorded. This finding has an explanation as a hormone more prevalent in males (testosterone) may play a role in increased risk-taking behavior, including substance abuse; some of the previous studies have reported a positive relationship between testosterone and aggressive behaviors [9]. In concordance with this study, Sadeghian et al., in a previous about the association of opium with coronary artery disease, 93.4 % of opium users were men and 5.6 % were women [10].

In this study, the mean age of study cases with confirmed opioid toxicity was 32 (SD ±14); this variation in age could be attributed to generational differences; different generations may have varying attitudes towards drug use and addiction. For example, older adults might have been exposed to opioids for pain management purposes; older adults are often prescribed more medicines than other age groups, resulting in an elevated likelihood of encountering potentially addictive substances. In contrast, younger demographics may exhibit a higher susceptibility to the recreational utilization of opioids [11]. This agrees with another study by Dydyk et al., discussing opioid use disorders, which revealed that Opioid-related fatalities are predominantly observed in individuals within the age range of 40–50 years. In contrast, heroin overdose incidents exhibit a higher prevalence among those aged 20–30 years [12].

This study showed that not working participants are more likely to be opioid abusers; participants who have primary education (low level of education) are more likely to be opioid abusers. A positive correlation between marital status and opioid abuse (unmarried participants are more likely to become opioid abusers) and socioeconomic status (participants with low socioeconomic status are more likely to be opioid abusers). This finding corroborates the research conducted by Carrière et al. in Canada, which disclosed elevated incidences of opioid intoxication among individuals with diminished income and educational attainment, those who were unemployed or disengaged from the labor market, as well as individuals residing in single-parent family structures [13].

Regarding the smoking status of study cases, the majority of study cases were smokers (84.3 %), and their mean age when starting smoking was 19 (SD ± 3.8). There was an association between smoking status and opioid abuse (smokers are more likely to be opioid abusers). Similarly, Sadeghian et al. findings indicated that the prevalence of cigarette consumption was significantly elevated among individuals who abuse opium, with 57 % of opium users engaging in smoking compared to merely 15 % of non-users [10]. Regarding the control group, more than half of them were working (70.6 %), (17.6 %) had university degrees, nearly half of them had average socioeconomic status (49 %), and most of them were nonsmokers (86.3 %). This strongly proves that opioid abuse is associated with smoking, unemployment, and low socioeconomic status.

More than half the cases abused opiates as a natural form of opioids (60.8 %), with positive opioid urine screening test n=31 (60.8 %). In comparison (37.3 %) were intoxicated with tramadol, with positive tramadol urine screening test n=18 (35.3 %) and positive urine screening for both tramadol and opioid n=2 (3.9 %). The most common mode of toxicity (86,3 %) was addict overdose. This agrees with another study by Eldin et al., including 87 cases admitted to the Intensive Care Unit (ICU) exhibiting substance abuse; group I (comprising cases involving tramadol and opioid substances) accounted for 67.87 % of the overall cases (n=59) [1]. On the contrary, Abd Allah et al. found that the prevalence of tramadol dependency among substance abuser patients who used one substance was as follows: tramadol 30.30 % (n = 100), heroin 11.52 % (n = 38), tramadol abuse was more prevalent than heroin, the difference between the two studies may be due to easy accessibility of tramadol in area of their study [14].

In this study, regarding the clinical staging of poison severity score, results show the classification of study cases with confirmed opioid toxicity according to poison severity score, metabolic system, nervous system, cardiovascular system, and respiratory system are most commonly affected. Metabolic system changes were mostly moderate grade II with more pronounced acid-base disturbance; 51 % presented with respiratory acidosis n=26. Regarding the nervous system, nearly half of the cases were moderate grade II (49 %) and presented with coma with an appropriate response to pain, brief apnea, dyspnea, agitation, seizures, and constricted non-reactive pupils. Another research by Mahmoud & Sarhan., which was conducted to recognize the incidence of acute toxicity of abused drugs in the Minya poison control center regarding acute toxicity of tramadol, showed similar results: the state of coma (58.6 %) represented the predominant clinical manifestation. In comparison, 40 % of the cases exhibited constricted pupils, and 57.1 % of the patients presented with acidosis [15].

Respiratory system changes vary from minor grade I (mild dyspnea) (41.2 %), moderate grade II (hypoxia requiring extra oxygen) (31.4 %), and severe grade III (pulmonary edema, pneumonia) (17.6 %). This can be explained by opioid analgesics, which have been shown to induce substantial ventilatory dysfunction, a marked decrease in oxygen saturation levels, and episodes of apnea shortly following their administration. Opioid-induced ventilatory Impairment (OIVI) is characterized by three principal clinical manifestations: diminished central respiratory drive, attenuation of supraglottic muscle tone, which may result in upper airway obstruction and reduced levels of consciousness [16]. On the contrary, Eldin et al. found that mechanical ventilation was required in almost half of the cases with tramadol and opiate toxicity (47.6 %); the difference could be related to the difference in delay time between cases in the two studies [1].

Regarding the cardiovascular system, the severity of symptoms varies from minor, moderate, and severe; more than half of cases showed moderate symptoms grade II (52.9 %) (sinus bradycardia, sinus tachycardia, prolonged QRS, myocardial ischemia, more pronounced hypotension), only (7.8 %) presented with severe symptoms grade III (myocardial infarction, shock, life-threatening ventricular arrhythmia). On the contrary, Eldin et al. found that CVS manifestations were less commonly recorded (24 %) in tramadol and opioid cases; the difference between the two studies could be attributed to the variability in individual responses [1].

Regarding cardiac profile assessment results, by local cardiac examination, (39.2 %) of cases with confirmed opioid toxicity had hypotension n=20, (13.7 %) of cases had murmur n=7, (3.9 %) of cases had irregular pulse n=2, (3.9 %) with absent peripheral pulsation n=2 compared with normal examination of the control group. In line with this study, Krantz et al. found that the cardiovascular manifestations associated with acute opioid receptor activation are extensively documented and encompass phenomena such as hypotension, orthostatic changes, syncope, and bradycardia. The mechanism of hypotension is predominantly facilitated through the activation of μ-opioid receptors [17].

Studies showed expanding evidence of endocarditis as a significant and growing health concern for people who inject drugs, represented as the main cause of murmur in those people [18], [19]. Similarly, Krantz et al. determined that the incidence of bacterial endocarditis characterized by valvular involvement and audible murmurs experienced a near doubling (from 15.2 % to 29.1 %) over five years, coinciding with the opioid crisis. Epidemic [17]. Giudice et al., on the contrary, showed that Peripheral pulses are usually normal [20].

In the current study, regarding electrocardiogram results, ECG changes were more prominent among cases 72.5 % n= 37 had ECG changes compared to the control group, the most common ECG changes were sinus bradycardia 51 % n=26, 13.7 % presented with hyperacute T wave, 9.8 % with sinus tachycardia, 9.8 % with ST-segment elevation, 5.9 % presented with hyperacute T wave, 2 % presented with irregular rhythm, 2 % presented with first-degree heart block, 3.9 % with wide QRS complex, 3.9 % with RB right bundle branch block.

This agrees with Salem et al., who found a significantly heightened prevalence of electrocardiogram abnormalities among individuals involved in substance use compared to those who abstain from these activities, with the abuse of heroin and tramadol recognized as contributing risk factors for the occurrence of atypical ECG findings [21]. In line with the current study, Badar et al. showed that the cardiovascular system is affected by opioid toxicity, and an increase in cardiovascular disorders, including acute coronary syndrome, congestive heart failure, arrhythmias, and QTc prolongation, has been linked to opioid overdose [22].

In agreement with Greenwald et al., studying the effects of heroin use on resting cardiovascular function, on a sample of 292, they found that participants exhibiting a supine heart rate of less than 60 beats per minute were classified as having sinus bradycardia. In comparison, those displaying a supine heart rate of less than 50 beats per minute were categorized as having profound bradycardia. In total, 37 % of participants demonstrated sinus bradycardia, whereas only 5 % were identified as having profound bradycardia. The occurrence of sinus bradycardia was significantly anticipated by the frequency of heroin usage days in the preceding month [23].

In agreement with this study's results about T-wave abnormalities, Salem et al., in previous research, revealed that tramadol abuse and heroin abuse are significantly associated with the odds of abnormal ECG findings, abnormalities detected among substance abusers in their study were T-wave and ST-segment abnormalities [21]. Similarly, Mostafavi et al. demonstrated that ECGs were totally normal in 48 (48 %) patients. Three patients suffered extensive myocardial infarction MI (anterior MI in all 3 cases). The ECGs in 49 patients showed ST depression, inverted T, or both [24].

Sadeghian et al., in a previous study, showed that an elevated consumption of opium correlates with an increased incidence of coronary artery disease (CAD). In contrast to individuals exhibiting dyslipidemia, hypertension, a positive family history, and advanced age, those who partake in opium are predisposed to a higher likelihood of developing CAD [10]. The elevated incidence of coronary artery disease (CAD) among individuals who use opium may be elucidated by the interaction of opioids within the hypothalamic region, where they inhibit the secretion of gonadotropin-releasing hormone (GnRH). This inhibition subsequently results in diminished levels of luteinizing hormone and follicle-stimulating hormone, culminating in a reduction of testosterone concentrations. Consequently, reduced testosterone levels are posited to augment the likelihood of developing coronary atherosclerosis. Furthermore, alternative physiological mechanisms may also serve as contributing factors [10].

On the contrary, Greenwald et al., studying the effects of heroin use on resting cardiovascular function on a sample of 292, concluded that heroin use was not a predictor for QRS duration, PR interval, P-wave duration, or ST-segment elevation [23].

Although sinus bradycardia in opioid users is commonly seen, in the current study, we found that about 9.8 % of cases presented with sinus tachycardia. Remskar et al. similarly reported sinus tachycardia in a 17-year-old female with no prior significant medical history who was admitted to the intensive care unit a few hours subsequent to an intravenous heroin overdose, exhibiting a state of unconsciousness, arterial pressure (AP) measured at 60 mmHg, and a systolic central venous pressure (CVP) of 21 mmHg, alongside a heart rate (HR) of 156 beats per minute. The electrocardiogram (ECG) revealed sinus tachycardia accompanied by nonspecific abnormalities within the ST-T segment in the precordial leads [25].

In this study, 2 % of cases presented with an irregular rhythm, 2 % presented with first-degree heart block, 3.9 % with wide QRS complex, and 3.9 % with RBBB right bundle branch block. Ventricular arrhythmia occurs secondary to hypoxia that occurs with opioid overdose as a result of respiratory depression; opioids act on the μ receptors and suppress the respiratory system [6], [26]. Riasi et al., on the contrary, studied electrocardiographic changes in children with acute opioid poisoning; a total of 85 children, 38.8 %, were poisoned with synthetic opioids. The most common ECG change was prolonged QT interval (>450 ms) in 3.5 % of cases. Other ECG changes were limited to 1 U wave formation (1.2 %) that was detected in a patient with methadone poisoning. Differences between the two studies may be due to differences in type of opioid abuse [3].

In this study regarding echocardiography assessment, out of 51 cases with confirmed opioid toxicity, 20 cases (39.2 %) were selected for echocardiography according to their clinical severity. Out of 20 cases that performed echocardiography, 13 cases (60 %) had normal ejection fraction EF, 7 cases (40 %) had abnormal ejection fraction, and no abnormality was detected in the control group. The lowest EF (25 %) was recorded in a 43-year-old male smoker and heroin addict with an ECG showing signs of anterior myocardial infarction. Abnormal regional wall motion 7.8 %, valvular abnormalities including tricuspid regurge TR 7.8 %, mitral regurge MR 5.9 %, dilated right ventricle and dilated right atrium 5.9 %, dilated left ventricle and dilated left atrium 2 %, infective endocarditis and vegetations 2 %. Salem et al., in line with our results, found that there was a significantly higher prevalence of echocardiographic abnormal findings among substance abusers, including opioids (53.7 %), in comparison to non-abusers (9.3 %) [21].

In agreement, Mostafavi et al., in a cross-sectional investigation involving one hundred cases of methadone overdose, revealed that the left ventricular ejection fraction (LVEF) varied from 25 % to 69 %. Furthermore, it was observed that all instances exhibiting a diminished LVEF were concurrently associated with significant coronary artery disease (CAD), with the exception of two patients who were identified as having Takotsubo cardiomyopathy [24]. Contrary to what Yildirim et al. observed, heroin use does not seem to have any effects on the left ventricular functions, with a subclinical reduction in the ejection fraction of the left ventricle in 20 heroin-dependent individuals [27].

Regarding segmental wall motion, Salem et al. found that abnormalities in segmental wall motion were frequently observed (18.5 %), often accompanied by acute coronary syndrome, and strongly correlated with substance abuse, including opioids. ^21^ On the contrary, Salem et al. showed that the most common abnormality detected in their study was diastolic dysfunction in 25.9 % of patients. ^21^In agreement, Yildirim et al. studied the effect of heroin on electrocardiographic parameters and showed that heroin use significantly raises the incidence of tricuspid and mitral valve defects [27].

Results of this study showed that regarding cardiac enzyme assessment, in most cases, 77.7 % with confirmed opioid toxicity who were investigated for troponin I had increased troponin levels; regarding CK-MB, more than half of cases had increased CK-MB 51 %.

In agreement with Gulpembe et al., a case report of a young man with opiate abuse, he was presented with very high cardiac markers Troponin I, creatinine kinase (CK), CK-MB at admission and follow-up [28]. Similarly, Sheibani et al., in another observational, prospective study, results showed that about 19 (7.7 %) patients had Troponin I levels of >0.1 ng/ml (positive), and 41 (16.7 %) had borderline levels of 0.019–0.1 ng/ml. Suppose the clinical context is incompatible with myocardial infarction caused by coronary artery disease. In that case, opioid exposure should be taken into consideration in individuals with high levels of high-sensitivity troponin of unknown origin [29].

This study revealed that out of the sociodemographic characteristics, sex and smoking status (being male and smoker) are more likely to be predictors of developing cardiac affection. Contrary to Maas & Appelman., according to statistics from the National Health and Nutrition Examination Surveys (NHANES), the prevalence of myocardial infarctions has grown in midlife (35–54 years old) women during the past 20 years. Still, it has decreased in males of comparable ages [30].

Results of this study revealed a significant association with the type of substance abused opiate abusers are more likely to develop cardiac affection), cardiac affection was highly associated with the kind of substance abused, 60 % among opiates-intoxicated patients, and 30 % among tramadol-intoxicated patients. Other parameters are not significantly associated with developing cardiac affection.

In agreement with Salem et al., multivariable binary logistic regression analysis for factors related to abnormal ECG findings showed that tramadol abuse and heroin abuse were significantly associated with the odds of abnormal ECG findings at p<0.05 [21]. Similarly, Gupta et al. showed that opium consumption was linked with postoperative atrial fibrillation AF following coronary artery bypass grafting and was considered to be a new predictor of AF; there is a greater prevalence of sinus tachycardia, sinus bradycardia, and AF following acute myocardial infarction in patients with opium addiction compared with non-opium addicts [26].

Regarding the poison severity score and its correlation with other variables, our results revealed significant associations with CK-MB levels and severity of nervous system manifestations according to the poison severity score (patients with severe CNS manifestations, such as coma, were suspected of having increased CK-MB levels). Simonovska et al., On the contrary, they tested their results in relation to the severity of symptoms according to poison severity score PSS and gender; in most of the subjects, the PSS score was moderate and severe with no differences between genders [2].

Regarding clinical outcome, results revealed that more than half of intoxicated cases had morbidities, while 25 % showed complete recovery, and 16 % died. The most common morbidities among cases with confirmed opioid toxicity were CNS depression 64.7 % were in a coma, 17.6 % had seizures, 39.2 % had a shock, 17.6 % developed non-cardiogenic pulmonary edema, and only 2 % of cases developed sepsis. The mean and SD of ICU stay length (hours) was 87.7 (49.8), while the duration of mechanical ventilation M.V. (hours) among cases with confirmed opioid toxicity mean (SD) was 72.1 (49.7).

The primary mechanism of shock could be attributed to the effects of acute opioid receptor stimulation leading to cardiovascular manifestations, including hypotension, orthostasis, syncope, and bradycardia. Hypotension is primarily mediated through mu-receptor vasodilatation, which is consequently linked with common adverse events such as peripheral edema, flushing, and palpitations [17].

Noncardiogenic pulmonary edema (NCPE) is common in opioid overdose, involving up to 50 % of acute overdoses and the majority of fatalities. Both hemodynamic data and fluid analysis confirm the edema to be noncardiogenic in nature. Most cases involve loss of consciousness, with presumptive hypoventilation preceding NCPE. The mechanism appears related to hypoxic stress-inducing pulmonary capillary fluid leak. No data conclusively support a receptor-mediated mechanism in NCPE, and naloxone has not been demonstrated to be of benefit [31].

Similarly, Eldin et al. found that cases with tramadol or/ opioid overdose admitted to the ICU revealed they were mainly related to opioids. Disturbed level of consciousness was present in almost all cases (96.61 %), mainly as grade II coma (45.76 %) and irritable coma (25.42 %); shock was found in 11.86 % of cases [1].

On the contrary, Eldin et al. found that a short ICU stay (one day or less) was noted in the majority of cases (77 %), and recovery was recorded in more than half of the studied cases (56.32 %). Overall, in-hospital mortality and morbidity were 11.49 % and 10.34 %, respectively. While morbidity was higher among those who stayed more than one day in ICU, mortality was recorded mostly during the first 24 hours. The unknown outcome was noted in 21.84 % of cases due to discharge against medical advice; short ICU stay was significantly noted among tramadol and opioids cases [1].

Results in this study revealed a significant correlation between Troponin-I, CK-MB, and ICU stay lengths; an increased length of ICU was associated with high levels of Troponin and CK-MB. Significant associations with Troponin-I (increased levels with death), CK-MB levels (increased levels with death), and ECG findings (sinus bradycardia and T wave inversion are associated with death).

Similarly, Sheibani et al. studied the prognostic value of high-sensitivity troponin in different clinical settings, indicating its relation to a worse prognosis. Opioid exposure/toxicity is a newly identified cause of elevated troponin I. Values >0.019 ng/ml, and particularly >0.0365 ng/ml, of troponin I predicted mortality in their sample. On the contrary, they did not find a significant relationship between ECG changes as QTc prolongation and mortality [29].

The discrepancy between our results and the studies mentioned above' results could be due to different study populations, variations in nationality, community habits and traditions, and differences in the type of opioid abuse.

## 5 Conclusion

Opioid abuse and acute opioid intoxication were more prominent among males, those not working, and smokers. Compared to the control group, more than half of them were working, and most of them were nonsmokers. The most common ECG changes among cases with confirmed opioid toxicity were sinus bradycardia followed by T wave inversion, the most prominent echocardiography abnormalities were abnormal regional wall motion and tricuspid regurge, cases had more elevated levels of CK-MB and Troponin I compared to the control group, The most common morbidities among cases were coma followed by shock and non-cardiogenic pulmonary edema; cardiovascular changes were more prominent in cases than control group, ECG, Echocardiographic and cardiac enzymes abnormalities have significant association with acute opioid intoxication.

## Sources of Funding

None.

## Acknowledgment of reviewer feedback

We express our gratitude to the reviewers for their insightful and constructive comments, which have significantly contributed to improving the quality and clarity of our manuscript. We have carefully addressed each point raised in the review process, ensuring that all necessary corrections and improvements have been made.

## Author statement

We confirm that this manuscript is original, has not been published previously, and is not currently under consideration by any other journal. All authors have approved the manuscript and agree with its submission to **Toxicology Reports**. We have also disclosed any potential conflicts of interest associated with this research.

## CRediT authorship contribution statement

**Mohamed Hemeda:** Writing – review & editing, Writing – original draft, Visualization, Validation, Supervision, Software, Resources, Project administration, Methodology, Investigation, Funding acquisition, Formal analysis, Data curation, Conceptualization. **Amany A Mostafa:** Writing – review & editing, Writing – original draft, Visualization, Validation, Supervision, Software, Resources, Project administration, Methodology, Investigation, Funding acquisition, Formal analysis, Data curation, Conceptualization. **Rawan M Ghaly:** Writing – review & editing, Writing – original draft, Visualization, Validation, Supervision, Software, Resources, Project administration, Methodology, Investigation, Funding acquisition, Formal analysis, Data curation, Conceptualization. **Heba Youssef Sayed:** Writing – review & editing, Writing – original draft, Visualization, Validation, Supervision, Software, Resources, Project administration, Methodology, Investigation, Funding acquisition, Formal analysis, Data curation, Conceptualization.

## Declaration of Competing Interest

The authors declare that they have no known competing financial interests or personal relationships that could have appeared to influence the work reported in this paper.

## Data Availability

Data will be made available on request.
